# Informing the development of an outcome set and banks of items to measure mobility among individuals with acquired brain injury using natural language processing

**DOI:** 10.1186/s12883-022-02938-1

**Published:** 2022-12-09

**Authors:** Rehab Alhasani, Mathieu Godbout, Audrey Durand, Claudine Auger, Anouk Lamontagne, Sara Ahmed

**Affiliations:** 1 School of Physical and Occupation Therapy, Faculty of Medicine, McGill University, 3655 Sir William-Osler, Montreal, QC H3G 1Y6 Canada; 2 Centre for Interdisciplinary Research in Rehabilitation of Greater Montreal, Montreal, Quebec Canada; 3 Department of Rehabilitation Sciences, College of Health and Rehabilitation Sciences, Princess Nourah Bint Abdulrahman University, Riyadh, Saudi Arabia; 4 Université Laval, Laval, Quebec, Canada; 5 Mila - Quebec Artificial Intelligent Institute, Montreal, Quebec Canada; 6 School of Rehabilitation, Faculty of Medicine, Université de Montréal, Montreal, Quebec Canada; 7 Institut universitaire sur la réadaptation en déficience physique de Montréal, CIUSSS du Centre-Sud-de-l’Île-de-Montréal, Montréal, Quebec Canada; 8 Jewish Rehabilitation Hospital, CISSS de Laval, Laval, Quebec Canada; 9 McGill University Health Center Research Institute, Clinical Epidemiology, Center for Outcome Research and Evaluation, Montreal, Quebec Canada; 10 Constance Lethbridge Rehabilitation Center, CIUSSS Centre- Ouest de l’Îile de Montreal, Montreal, Quebec Canada

**Keywords:** Acquired brain injury, Mobility, Natural Language Processing, Machine Learning, Core Outcome Set, Item banks

## Abstract

**Background:**

The sheer number of measures evaluating mobility and inconsistencies in terminology make it challenging to extract potential core domains and items. Automating a portion of the data synthesis would allow us to cover a much larger volume of studies and databases in a smaller fraction of the time compared to the usual process. Thus, the objective of this study was to identify a comprehensive outcome set and develop preliminary banks of items of mobility among individuals with acquired brain injury (ABI) using Natural Language Processing (NLP).

**Methods:**

An umbrella review of 47 reviews evaluating the content of mobility measures among individuals with ABI was conducted. A search was performed on 5 databases between 2000 and 2020. Two independent reviewers retrieved copies of the measures and extracted mobility domains and items. A pre-trained BERT model (state-of-the-art model for NLP) provided vector representations for each sentence. Using the International Classification of Functioning, Disability, and Health Framework (ICF) ontology as a guide for clustering, a *k*-means algorithm was used to retrieve clusters of similar sentences from their embeddings. The resulting embedding clusters were evaluated using the Silhouette score and fine-tuned according to expert input.

**Results:**

The study identified 246 mobility measures, including 474 domains and 2109 items. Encoding the clusters using the ICF ontology and expert knowledge helped in regrouping the items in a way that is more closely related to mobility terminology. Our best results identified banks of items that were used to create a 24 comprehensive outcome sets of mobility, including Upper Extremity Mobility, Emotional Function, Balance, Motor Control, Self-care, Social Life and Relationships, Cognition, Walking, Postural Transition, Recreation, and Leisure Activities, Activities of Daily Living, Physical Functioning, Communication, Work/Study, Climbing, Sensory Functions, General Health, Fatigue, Functional Independence, Pain, Alcohol and Drugs Use, Transportation, Sleeping, and Finances.

**Conclusion:**

The banks of items of mobility domains represent a first step toward establishing a comprehensive outcome set and a common language of mobility to develop the ontology. It enables researchers and healthcare professionals to begin exposing the content of mobility measures as a way to assess mobility comprehensively.

**Supplementary Information:**

The online version contains supplementary material available at 10.1186/s12883-022-02938-1.

## Background

Acquired Brain Injury (ABI), including traumatic brain injury (TBI) and stroke, is most prevalent cause of disability globally [1–3]. According to the World Health Organization, the global incidence of all-severity TBI is estimated at 69 million people, while 15 million people suffer a stroke worldwide each year [4–6]. Among the 1.5 million Canadians with ABI that go through the care continuum annually; over 60% report ongoing restrictions in mobility and participation in societal roles [5]. Individuals with ABI can continue to experience improvements in mobility to improve participation and well-being when rehabilitation intervention can be offered in the community. However, the often the accessibility to the rehabilitation pathway is complex and time-consuming [7–9]. Thus, the effect on individuals, health care systems, and society suggest a greater need to focus attention on the long-term consequences, management, and rehabilitation of people with ABI [10].

Mobility is a multidimensional construct defined through both theoretical and empirical approaches. From a theoretical point of view, mobility has frequently been defined in terms of life-space frameworks as the ability to move oneself, including any age, within environments that expand from one's home to the neighbourhood and regions beyond [11–18]. Mobility is influenced by five vital inter-related determinants, including physical, environmental, cognitive, psychosocial and financial influences [14], and this is reflected in the International Classification, Functioning, Disability, and Health framework (ICF) core set [19]. Empirical studies have also focused on the effects of the built environment including technological parts, such as mobility aids, on community mobility [20, 21].

Selection of a suitable outcome measure is critical to accurately characterize and monitor changes in mobility during rehabilitation interventions for adults with ABI [22]. However, selection can pose a challenge to both researchers and clinicians as the range of outcome measures available in the clinical research literature is vast, and distinctions between them are often not clear [23, 24]. Researchers and clinicians also need to consider the content of measures and whether the domains evaluated match research and clinical objectives. Multifaceted assessments of mobility among individuals with ABI can assist in the development of individualized rehabilitation treatment plans that could enhance patients’ global health status and allow the evaluation of the long-term effectiveness of interventions [25, 26].

Mobility is commonly assessed through performance-based measures (e.g., walking tests) or clinician-reported outcomes (e.g., Disability Rating Scale) [27–29]. Although these measures capture some aspects of functional capacity, they are not comprehensive enough to evaluate patients’ perspective on their function, nor the effects of their limitations on everyday life. In the last 20 years, advances in measurements have brought to the research and clinical practice the assessment of quality of life through patient-reported outcome (PRO) measures [30, 31]. Mainly, the National Institutes of Health’s Patient-Reported Outcomes Measurement Information System (PROMIS), the Quality of Life in Neurologic Disorders (Neuro-QoL) and the Traumatic Brain Injury Quality of Life (TBI-QOL) initiatives have pioneered the development of PRO measures [30–33]. These initiatives have resulted in the development of measures that allow comparison across conditions over time, testing of all levels of function with one measure, reduce the administration of irrelevant items to a given individual, and minimize testing time by reducing the overall number of items administered through short forms [26, 32, 33]. Although these initiatives have made great advances in general population and neurological population assessment, neither measurement system alone can capture the multi-dimensionality of mobility among individuals with ABI.

Core Outcome Sets (COS) developed by researchers and patients allow interventions to be evaluated by using an agreed-upon set of outcomes that can be compared across studies, and clinical care programs and settings. A COS includes measures, tools, and endpoints to assess a minimum list of impacts and demonstrate changes. The PROMIS (www.nihpromis.org, March 16, 2021) is charged with developing improved PROs applicable to all areas of chronic illness and involving several domains such as physical functioning and disability. PROMIS is the most ambitious approach yet to these issues [34–36]. In simplest terms, PROMIS seeks to employ the best items in the best ways [34–36] with a focus on items that are most relevant to study endpoints in clinical trials and observational studies. Optimal instrument development requires item improvement, yet systematic approaches to the advancement of improved items need to ensure items have full coverage of the construct of interest, and adjust item banks; if data supports that a given item is problematic, it is removed or revised to increase its relevance and clarity.

Compared to traditional manual consensus, utilizing machine learning (ML) helps researchers to develop item banks more efficiently and synthesize literature that manually is nearly impossible. ML is a subset of Artificial Intelligence that enables computers to learn without being explicitly programmed with predefined rules [37]. In the rehabilitation sciences, building computer programs that can extract and process knowledge from text documents at a level that is usable by experts in the domain requires several elements that can generally be associated with intelligence [37, 38]. This predictive ability enables ML to handle massive datasets with efficiency and accuracy. ML algorithms are categorized into supervised learning, unsupervised learning, and reinforcement learning [39]. Natural language processing (NLP) is unsupervised ML that focuses particularly on textual data/info/input [40]. The ultimate objective of NLP is to read, decipher, understand, and make sense of the human languages in a manner that is valuable [40]. For example, a key feature of NLP is to generate embeddings for extents of text [41]. Text embeddings can be used to ease learning in downstream tasks and naturally encode similarity whether it is on the word-level or sentence-level [42].

Properly classifying content from mobility measures is needed to identify relevant texts. Often, this process relies on pre-defined static vocabularies that describe the mobility domains. To understand knowledge evolution, the initial system vocabularies should evolve in an automatic way in order to correctly reflect and evolve our understanding about mobility. Our goals for this work were to identify optimal domains by extracting and classifying items from published research of mobility measures. We did this using NLP technique to create sentence embeddings to inform the mobility ontology. NLP was selected as an approach robust enough to develop preliminary banks of items of mobility that used to evaluate each domain in a comprehensive outcome set of mobility among individuals with ABI.

### Objective

While using NLP, we aimed to: (1) identify a comprehensive outcome set of mobility, and (2) develop preliminary banks of items of mobility among individuals with ABI.

## Methods

### Step 1: Item selection process

To develop preliminary banks of items of mobility among individuals with ABI, we conducted a comprehensive umbrella review of mobility measures among individuals with ABI [43] following the 10 steps of the Consensus-based Standards for the Selection of Health Measurement Instrument (COSMIN) guideline for systematic reviews [44]. Subsequently, we conducted focus group discussions among clinicians and individuals with ABI and their caregivers to identify factors limiting or enhancing mobility that need to be considered when evaluating mobility [45].

1.1. Search strategy: A comprehensive search of the literature was performed using electronic databases of Ovid MEDLINE, CINHAL, Cochrane Library and EMBASE from 2000 to March 2020. The search was conducted in collaboration with a health sciences librarian to ensure that the review included the appropriate and necessary keywords. A combination of Medical Subject Headings (MeSH) terms, subject headings and/or key words was used. Three groups of terms were generated describing: (1) the population “acquired brain injury” AND; (2) the outcome measure “mobility” AND; (3) the psychometric properties. Terms within each group were combined with the Boolean operator ‘OR’. Because the search included different types of studies, the search was narrowed by filtering the search specifying the type of studies including systematic review, review, and meta-analyses. This filter has been used to avoid missing important information related to mobility measures.
1.2. Select abstracts and full text articles: Inclusion of articles was based on the agreement between two independent reviewers. Disagreements were resolved by discussion and consensus. If required, a third reviewer was consulted. The reference list of the articles included for the full text screening was also hand-searched for additional identification of relevant articles. The Preferred Reporting Items for Systematic Reviews and Meta-Analyses (PRISMA) flow diagram [46] was used to guide the selection process.
1.3. Eligibility criteria: Inclusion criteria for the umbrella review were reviews published in peer-reviewed journals, including individuals with ABI (Stroke, traumatic brain injury) over 18 years old. They report a clear objective of identifying measures of mobility. They include either multiple or single measure(s) of mobility including different sources of information (i.e., clinicians, patients, and technology). The exclusion criteria were reviews investigating effectiveness of interventions or treatments, monitoring recovery, focusing on diagnostic screening or prognosis, clinical commentaries, case reports, non-human studies and grey literature. Also, systematic reviews not published in English or French were excluded.
1.4. Data extraction: Two independent reviewers extracted the measures from the reviews, retrieved copies of measures from the literature, and included the non-covered measures identified from the focus groups. They extracted measures’ domains and items manually, to avoid missing relevant information. Also, they added mobility domains (i.e. factors) identified from the focus groups.

### Step 2: Data cleaning

The data cleaning process ensures that the domains and items are consistent and accurate. The following steps were applied to the processed terms using Microsoft office Excel 2010 (Additional file 1: Appendix 1 presents the functionalities that used in this process):

2.1. Export to .CSV file and create a backup copy of the original data in a separate spreadsheet.
2.2. Remove duplicate rows: we filtered for unique values first to confirm that the results were what we wanted before removing duplicate rows.
2.3. Correct spelling mistakes: lexical matching requires correction of spelling mistakes. For example, behaviour becomes behavior; practise becomes practice; neighbour becomes neighbor, and so on.
2.4. Changing the case: all the uppercase letters were converted to lowercases letters.
2.5. Extend acronyms and abbreviations to their full form: because they caused mismatches in the string-matching process, acronyms and abbreviations were removed, such as 6MWT becomes six-minute walking test, BI becomes brain injury, and so on.
2.6. Fixing numbers and number signs.
2.7. Remove white spaces, non-printing characters, typos, punctuations from the sentence, and use underscore (_) instead of dash (-).

### Step 3: The proposed model

**Fig. 1:**
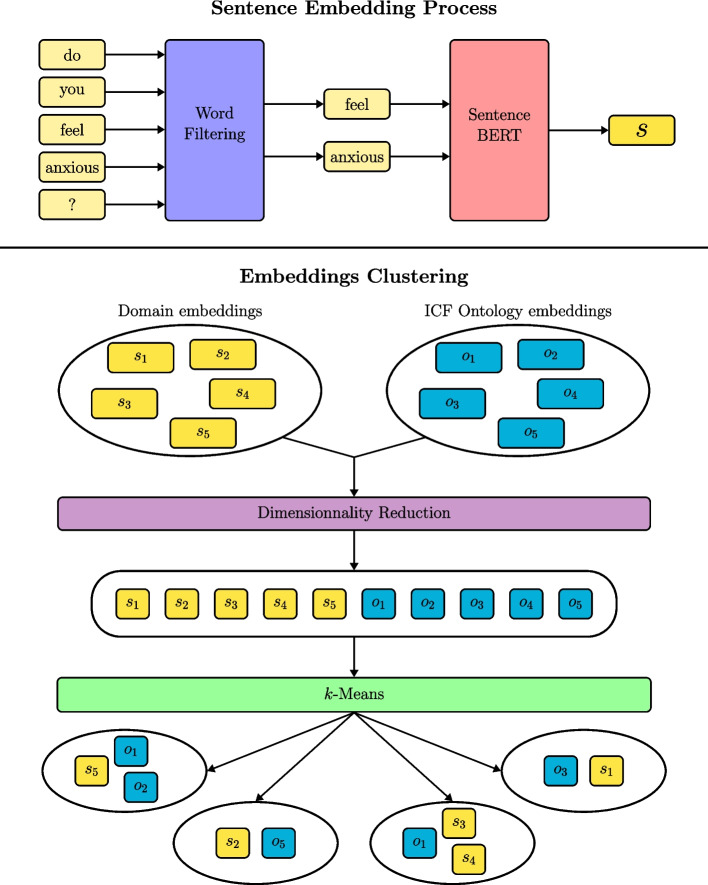
An overview of the proposed model

Figure 1 presents an overview of the proposed model that was used to analyze the data using the NLP technique. Python 3.0 Release was used to analyse the data. All the process details are described below:

3.1. For each mobility item, we first applied a word filtering that was hypothesized to remove noise from the word groups. The different filters considered were: the absence of filter; filtering all words with fewer than 4 letters; filtering words contained in a public stop-words dictionary; and filtering words based on their occurrence, where words seen too often in the dataset were removed from their group.
3.2. Generate database on neural network processing of 15 million articles on mobility and ABI using Mesh terms from Pubmed to train our Fast-Text embeddings.
3.3. Using the pre-trained Bidirectional Encoder Representations from Transformers (BERT) model (state-of-the-art model for NLP) [47], we created sentence embeddings, in which the collected sentences (items) were mapped to vectorial representations, i.e. vectors of real numbers (https://www.sbert.net/index.html, March 16, 2021).
3.4. Vectorial representations generated from Sentence-BERT model included 768 dimensions. These dimensions are inefficient for distance-based clustering, as the usual distance metrics suffer from the curse of dimensionality and sentence clustering becomes very difficult [48]. To ease computation, we applied a Principal Component Analysis (PCA) decomposition [49, 50] from the scikit-learn library (https://scikit-learn.org/stable/about.html#citing-scikit-learn, March 16, 2021) [51] to reduce the sentence embeddings’ dimensions.
3.5. The ICF terms, extracted from the ICF ontology (https://bioportal.bioontology.org/ontologies/ICF, March 16, 2021), were used to focus the embedding clustering on mobility and mobility determinants. The ICF terms went through the same pipeline of word filtering, Sentence-BERT and dimensionality reduction.
3.6. The *k*-means algorithm [52] was applied to all collected sentence embeddings to retrieve clusters of similar sentences.
3.7. To evaluate the quality of the resulting clusters, a Silhouette score [53, 54] was used. A Silhouette score is a clustering metric ranging between -1 to 1, and based on inter- and intra-cluster distances. A high Silhouette score means that sentences in a given cluster are similar and that different clusters are distinct. A Silhouette score can be used in our case, but evaluating the quality of the model was limited in terms of sentence embeddings, as the vectorial distance between sentences in one cluster were not well fitted to mobility-related proximity. Therefore, we used the Silhouette score to filter out promising clusterings and relied on expert input to select the final clustering.
3.8. We employ a grid search strategy to generate numerous clusterings from a range of key hyperparameters in our method. Namely, we searched over the following hyperparameter values:
1. *k* value in *k*-means, ranging from 4 to 40;
2. four (4) word filtering methods, listed in 3.1;
3. target dimension after PCA reduction, taking values in [5, 10, 25, 50];
4. total weight attributed to ICF terms in the *k*-means clustering, taking values in [0.0, 0.1, 0.25, 0.5].

We generate a clustering for every combination (*n*=592) of the above hyperparameter values. We retain only the 10 best clusterings according to an automatic heuristic, described in section 4. An expert then goes over the 10 retained clusterings and selects the most relevant one for further analysis. We argue that this two-step procedure is required by the intrinsic difficulty of the clustering evaluation task. Indeed, while the automatic heuristic filter first eliminates clusterings that only weakly correlated, i.e., underfitting, the expert decision at the end detects clusterings that have good correlation metrics but low relevance with the overall objective, i.e., overfitting, Underfitting and overfitting commonly arise in unsupervised settings such as ours due to the lack of ground-truth labels to assess the true performance of the model.

3.9. The above steps resulted in sentence clustering that was then analyzed by 4 experts (RA, CA, AL, SA), who reviewed the top 30 sentences (items) in each cluster following agreed-upon criteria, including: remove ambiguous, vague and parallel items; clarify items by adding or removing needed words; and label each item to an agreed-upon domain. The expert annotations were then used to fine-tune the Sentence-BERT model towards more meaningful mobility-related sentence embeddings. The final clustering respected expert annotations of 80 % F1-Score [55, 56].

### Step 4: Preliminary banks of items selection process

**Fig. 2:**
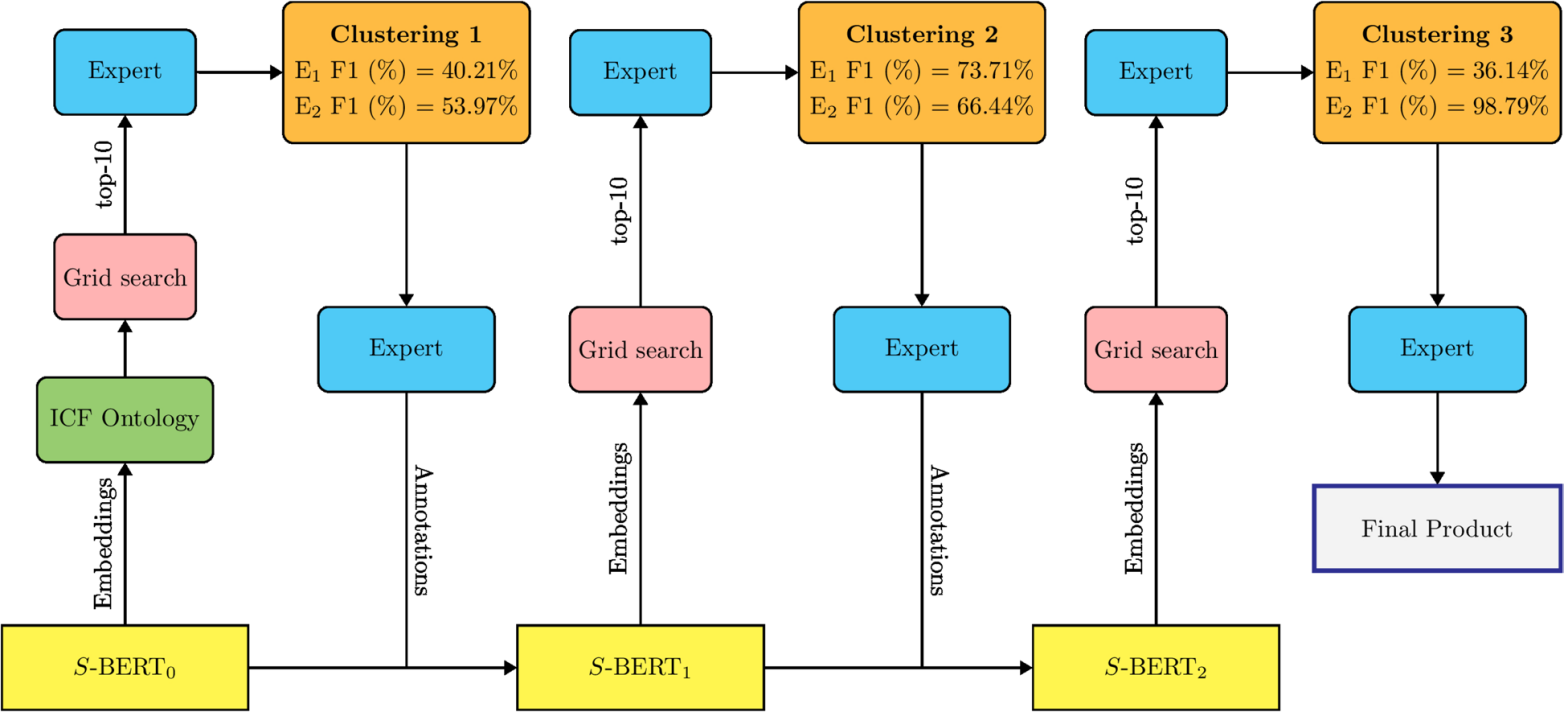
The iterative improvement process for preliminary item bank process. The process began with an initial Sentence-BERT model and relied heavily on the ICF ontology to produce a good enough first clustering. At each step, a grid search was collected over a wide range of hyperparameter values and a best clustering was retained according to automatic heuristics and human evaluation. After each clustering, expert annotations were collected to improve the Sentence-BERT model and yield better clusterings. We report the F1 score of each clustering with respect to the first and second expert annotations, respectively named E_1 and E_2. Here, E_2 is the most reliable metric, as it associates items with adequate labels, while E_1 associates item pairs with whether or not they belong together. By nature, E_1 penalizes having a large number of clusters, as can be seen on the third clustering's score. Also note that both E_1 and E_2 are not exact metrics, as, for instance, the third clustering still required heavy finetuning by experts to yield a satisfying Core Outcome Set despite the near-perfect E_2 score.

The most critical part of our proposed model is the sentence embedding process. The pre-trained Sentence-BERT model was used to produce semantically accurate embeddings (Fig. 2). To ensure the quality of evidence, the following was done:

4.1. First iteration: a small subset of mobility items was analyzed by the Sentence-BERT model using the ICF terms from the ontology as a guide. At this step, the automatic heuristic retained for filtering out the clusterings was the Silhouette score, due to the lack of automatically applicable human knowledge. The analysis yielded sentences that were correctly and incorrectly clustered. This information was used by the experts to create relations for sentence pairs that should or should not be clustered together.
4.2. Second iteration: the relation for sentence pairs that were extracted from the first iteration was used as a training example to fine-tune the sentence-BERT model. The automatic heuristic employed was the accuracy metric on the binary classification relations identified by the experts at the end of step 4.1. The resulted clusters from the second iteration were analyzed again by the experts who grouped hundreds of items by labelling them to an agreed-upon domain.
4.3. Third iteration: final results were obtained by fine-tuning the sentence-BERT model again with the newly expert knowledge. For this step, the automatic heuristic was the accuracy metric on the expert-identified binary relations from step 4.1 and domain-classification relations from step 4.2. The resulting best cluster consisted in 26 unified clusters of items.

## Results

### Search results

**Fig. 3:**
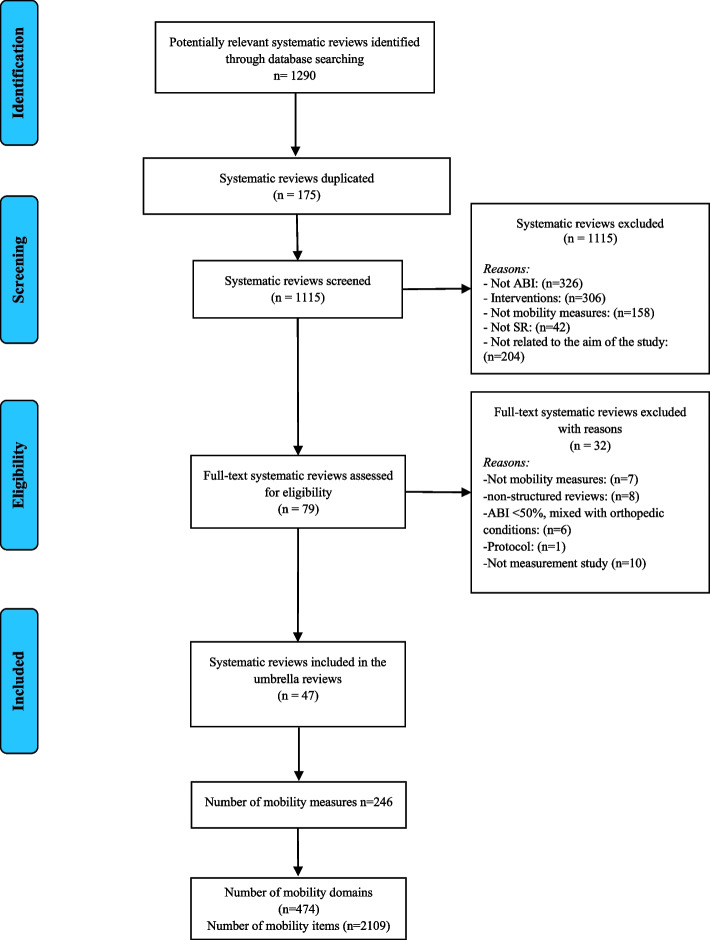
PRISMA flow diagram

The search strategy yielded a total of 47 reviews that met the eligibility criteria and were included [27, 57–102]. 246 copies of mobility measures were retrieved, and from these 474 mobility domains and 2109 mobility items were extracted. Figure 3 presents the PRISMA flow diagram, including the selection process and the reasons for exclusion.

### Identification of mobility outcome set and preliminary banks of items

Table 1 shows the hyperparameter values of the retained clustering for steps 4.1 to 4.3. Initially, our best grouping according to Silhouette score and expert knowledge resulted in 26 clusters. The experts reviewed each cluster of items and only included relevant and clear items. Duplicates (*n*=267), ambiguous parallel items (*n*=97), and fewer than 2 words items (*n*=134) were removed, resulting in 1611 out of 2109 items. In addition, among the 1611 items, 245 (15%) items were considered as outliers, as they did not fit well enough within their cluster. Also, seven clusters were identified as outliers, as they included items labelled to more than one domain. Results from the 26 clusters showed that fifteen clusters had no outliers; six clusters contained 5% to10% outliers; and ten clusters contained > 10% outliers.

**Table 1:** Grid search results for each of the three clusterings

Clustering	***K***^**a**^	Word filtering	PCA dimension	ICF weight	Silhouette score
Clustering 1	5	Words present >= 20 times	10	0.5	0.66
Clustering 2	6	None	50	0.5	0.65
Clustering 3	26	None	50	0	0.69

^a^Represent the best K values for Clustering 1, 2 and 3

**Fig. 4:**
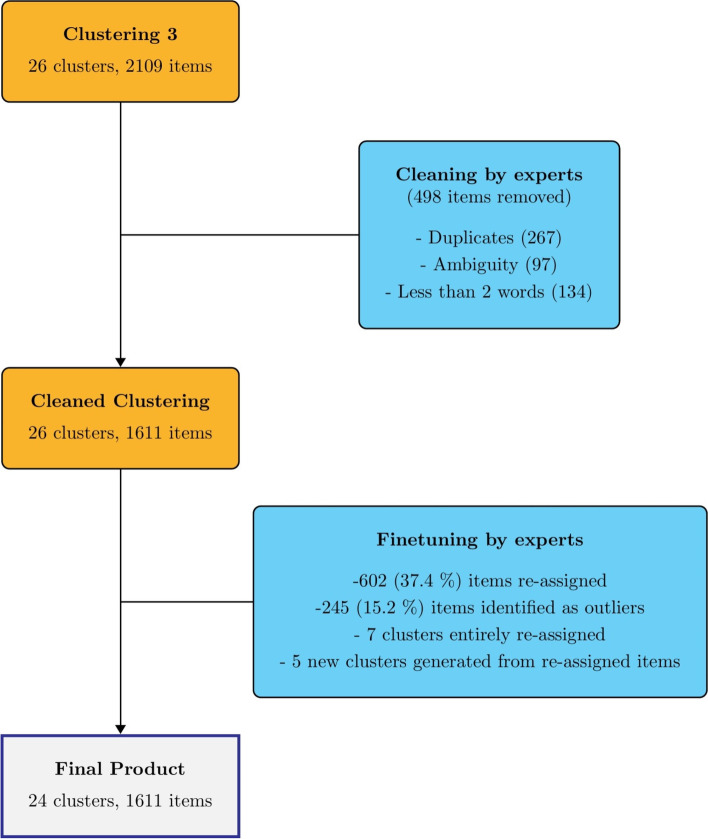
Identification of mobility Core Outcome Set and preliminary item banks from the third final Clustering. In the fine-tuning step, items were considered outliers when they did not match well enough with the cluster they were in (clustering inaccuracy). Re-assigned items are items that changed cluster between the Cleaned Clustering and the Final Product. Re-assigned items include outliers but also items that were part of a large cluster that was split to make smaller and more precise clusters

After extensive discussion, experts decided not to eliminate outliers which are not filtered by the algorithm, clusters labelled to more than one domain, and to manually reassign them to the fitted clusters. Additionally, five new clusters were generated from outliers not filtered by the algorithm. Overall, 602 (37%) of the items were reassigned in the fine-tuning process resulting in 24 preliminary comprehensive outcome set of mobility, namely: Upper Extremity Mobility, Emotional Functions, Balance, Motor Control, Self-care, Social Life and Relationship, Cognition, Walking, Postural Transition, Recreation and Leisure Activities, Activities of Daily Living, Physical Functioning, Communication, Work/Study, Climbing, Sensory Functions, General Health, Fatigue, Functional Independence, Pain, Alcohol and Drugs Use, Transportation, Sleeping, and Finances (Fig. 4 and Table 2). Also, we define the comprehensive outcome set of mobility conceptually based on the ICF and Webber’s frameworks in Table 3.

**Table 2:** Overview of the final item banks identification from Clustering 3

Name	Initial size	Outliers removed (%)	Outliers reassigned (%)	Final size
Upper Extremity Mobility	126	0 (0)	82 (39)	**208**
Emotional Function	119	37 (31)	93 (53)	**175**
Balance	125	32 (26)	57 (38)	**150**
Motor Control	131	31 (24)	10 (9)	**110**
Self-care	89	5 (6)	24 (22)	**108**
Social Life and Relationship	91	0 (0)	12 (12)	**103**
Cognition	55	3 (5)	40 (43)	**92**
Walking *(New)*	0	0 (0)	92 (100)	**92**
Postural Transition	107	32 (30)	15 (17)	**90**
Recreation and Leisure Activities	85	7 (8)	7 (8)	**85**
Activities of Daily Living	64	4 (6)	15 (20)	**75**
Physical Functioning	55	6 (11)	18 (27)	**67**
Communication	27	0 (0)	25 (48)	**52**
Work/Study	22	0 (0)	8 (27)	**30**
Climbing *(New)*	0	0 (0)	28 (100)	**28**
Sensory Function *(New)*	0	0 (0)	22 (100)	**22**
General Health *(New)*	0	0 (0)	20 (100)	**20**
Fatigue	13	0 (0)	19 (59)	**32**
Functional Independence	19	12 (63)	11 (61)	**18**
Pain	20	4 (20)	0 (0)	**16**
Alcohol and Drugs Use	14	0 (0)	0 (0)	**14**
Transportation	10	0 (0)	3 (23)	**13**
Sleeping *(New)*	0	0 (0)	13 (100)	**13**
Finances	10	0 (0)	1 (9)	**11**
*Removed*	127	127 (100)	0 (0)	**0**
*Removed*	121	121 (100)	0 (0)	**0**
*Removed*	69	69 (100)	0 (0)	**0**
*Removed*	34	34 (100)	0 (0)	**0**
*Removed*	21	21 (100)	0 (0)	**0**
*Removed*	41	41 (100)	0 (0)	**0**
*Removed*	16	16 (100)	0 (0)	**0**

*All manipulations were done manually by experts to yield the most coherent Core Outcome Set. Clusters who were completely reassigned to other clusters are marked *Removed* and clusters who only contain reassigned items are marked *(New)*.

**Table 3:** The comprehensive Core Outcome Set of mobility defined conceptually based on the International Classification of Functioning, Disability, and Health, and Webber’s frameworks

Cluster number and name	Definition
1. Upper Extremity Mobility	Defined as the ability to reach or rise up an object from one place to another, and perform the coordinated actions of handling, picking up, manipulating and releasing objects using one's hand, fingers and thumb.
2. Emotional Functions	Defined as mental functions related to the feeling including depression, anxiety and anger
3.Balance	Defined as the ability to maintain the body position within the base of support with minimal postural sway.
4. Motor Functions	Defined as functions associated with motor control and coordination of voluntary movements.
5. Self-care	Defined as the ability to caring for oneself, washing and drying oneself, dressing, eating and drinking, and looking after one’s health.
6. Social life and Relationship	Defined as the ability to carrying out the actions and tasks required for basic and complex interactions with people in a contextually and socially appropriate manner to engage in organized social life in community, social and civic areas of life.
7. Cognition	Defined as specific functions of the brain including memory and executive functions.
8. Walking	Defined as the ability to move along from point A to point B including, walking short or long distances; walking on different surfaces; and walking around and over obstacles.
9. Postural Transition	Defined as the ability to move from one surface to another without changing body position such as moving from a bed to a chair.
10. Recreation and Leisure Activities	Defined as the ability to engage in any form of play such as going to art galleries, museums, or cinemas for pleasure.
11. Activities of Daily Living	Defined as the ability to carrying out everyday actions and tasks including acquiring a place to live, preparing meals, household cleaning and repairing.
12. Physical Functioning	Defined as the ability to do various activities that require increasing degrees of strength and endurance.
13. Communication	Defined as specific features of communicating by speaking or carrying on conversations, comprehending and comprehension.
14. Work/Study	Defined as the ability to engage in all aspects of work including seeking employment and getting a job, doing the required tasks or studies to get the job.
15. Climbing	Defined as the ability to move upwards or downwards over different surfaces such as climbing stairs
16. Sensory Functions	Defined as functions of sense including vision, auditory, smell, touch and taste.
17. General Health	Defined as the status of complete physical, mental and social well-being.
18. Fatigue	Defined as functions related to respiratory and cardiovascular capacity for enduring physical exertion.
19. Functional Independence	Defined as the ability to perform an activity with no or little help from others.
20. Pain	Defined as an unpleasant feeling that indicates potential or actual damage to some body structure.
21. Alcohol and Drug use	Defined as substances that are harmful use for the mental
22. Transportation	Defined as using transportation to move around such as being driven in a car.
23. Sleeping	Defined as a characteristic physiological change accompanied by general mental functions of intermittent, reversible and selective physical and mental disengagement from one’s immediate environment.
24. Finances	Defined as products, such as money which serve as an exchange for labour, goods and services.

## Discussion

In this study, we identified a comprehensive outcome set of mobility and developed preliminary banks of items of mobility, for use in evaluating mobility among individuals with ABI, using NLP. We supported that it is possible to use a variety of existing instruments of mobility to build preliminary banks of items with promising properties using NLP. Although the PROMIS physical functioning item bank was found to be unidimensional, Mobility was constructed to represent a sub-domain of physical functioning to be used among individuals with chronic illnesses [30, 31, 103]. This study identified 24 preliminary banks of items of mobility, which need to be used to evaluate each domain in a comprehensive outcome set of mobility among individuals with ABI.

Improved outcome measures can substantially enhance clinical research and make the research process more efficient. Clinical trials may require fewer subjects, and greater assurances may be given that the perspectives of the patient are included. The goal of this work was to construct comprehensive mobility tools. Previous studies have shown that better items obtained from large item banks for relevant and clear items that can be understood and are considered important to patients, with less floor and ceiling effects, standardised time frames, content, and response options to improve item structure and wording [26, 32, 33]. The identified banks of items are required for researchers and health care professionals to compile and compare common mobility outcomes and items from centre to centre or client to client, directly influencing the identification and implementation of best practices [104].

An understanding of the nature and severity of mobility among individuals with ABI is needed, in order to develop effective individualized treatment plans and to compare different interventions. This requires a comprehensive assessment of impairments, activity limitations, and participation restrictions. The intervention plan varies depending on the patients' personal context, goals, and the complex interplay of the factors that influence mobility [14, 105]. This work provided a preliminary comprehensive outcome set of mobility from all possible sources, and mapped the constructs measured to the ICF. Results of this study will be used in future as part of an agreed-upon consensus of mobility COS, and the Delphi approach will be administered to achieve [106–108] expert consensus (i.e., clinicians and individuals with ABI and their caregivers), to examine mobility COS, to assess experts’ views on importance, clarity, and relevance of the domains and items of mobility, to unify the language of measuring mobility among individuals with ABI, and standardise measures used across clinical sites and studies.

In the rehabilitation sciences, developing NLP algorithms that can extract and process knowledge from text documents at a level that is usable by experts in the domain requires several elements that can generally be associated with intelligence [37, 38]. Throughout the experiments, it became clear that expert knowledge was the key factor in obtaining more accurate clustering. In the beginning, no expert knowledge was used and the best architecture artificially incorporated expert knowledge by requiring adding the ICF terms and to filter words in a sentence. The resulting clusters were also hard to evaluate automatically due to the poor quality of the pre-trained sentence-BERT embeddings for mobility-related tasks. The incorporation of expert knowledge gradually improved the quality of the resulting clusters. At the same time, the more information used allowed the sentence-BERT model to be further fine-tuned, gradually reducing the need to insert artificial knowledge in the procedure. Namely, on the final iteration, the best performing architecture did not filter words and did not require ICF terms. This shows that with iterations and fine-tuning of sentence embeddings, models improve in capturing the added expert knowledge. We note that our finetuning approach can be seen as an active learning finetuning process of a language model, as was already proposed for image caption classification for instance [109].

Step 2 was important in ensuring an item format that is consistent and coherent with the Sentence-BERT model’s input requirements. We however note that, while most of the tasks were done manually in our study, step 2 could be done entirely automatically. Since the nature of the study is to leverage NLP to increase the efficiency in generating outcome set, we believe automating step 2 would be a straightforward and important task in future iterations.

The use of item response theory (IRT) and computerised adaptive testing (CAT) is important in our next steps to provide item hierarchy and calibrate the items on a linear scale, respectively [110, 111]. IRT models incorporate both the characteristics of items and characteristics of individuals and calculate the probability of a positive response, to classify items for each person [35, 112, 113]. CAT is a specific kind of computer-based testing that asks questions extracted from larger pools of items covering a wider range of items difficulty to provide a more precise way to decrease questionnaire burden [35, 112, 113]. Moreover, IRT can quantitatively estimate the properties of each item and eliminate poor items to optimise the matching of items for each patient using CAT applications.

### Lessons Learned

“*Shared language is important in leading adaptive change. When people begin to use the same words with the same meaning, they communicate more effectively, minimize misunderstandings, and gain the sense of being on the same page, even while grappling with significant differences on the issues* [114]” One of the barriers to implement a COS of mobility to use among individuals with ABI has been the lack of a comprehensive common language describing domains of mobility in the healthcare professions. This gap of a common language prevented the development of a classification system of representative knowledge (i.e., ontology) that would allow the experts to make decisions related to tailored intervention plans among individuals with ABI. We therefore began this robust methodology using NLP with the goal of establishing preliminary banks of items of mobility that could be mapped within the continuum of care.

Lessons learned from this work include: First, NLP techniques require human annotations to thrive, as the work clearly indicated that expert knowledge was the key factor in obtaining more accurate clustering. Second, some measures included irrelevant and ambiguous items and we were able to examine and eliminate them. Third, the provided banks of items of mobility considered other item banks not identified in the literature search such as PROMIS. Toward that end, final consensus on a COS and banks of items of mobility needs to incorporate input from all stakeholders. Such item banks will provide a solid foundation to develop a commonly used ontology to inform selection of mobility outcomes and classification of mobility terms in digital health solutions and electronic medical records.

### Limitations

During the process of retrieving copies of measures, we faced some challenges related to some of technology-based and performance/clinicians measures. These challenges include: the difficulty of retrieving some technology-based measures such as actical, actigraph, motionlogger, goniometers, caltrac accelerometer, gyroscopes, magnetometer and sensewear pro 3 armband; the domains and items for some technology-based measures (such as Global Positioning System (GPS)); and for some performance/clinicians measures (such as gait speed, six minute walking test, timed up and go test, and manual functional test) were hard to extract.

While our methodology improved overall performance of the model, we note the following limitations in relation to the automatic NLP evaluation: traditional clustering metrics like the Silhouette score are only barely useful when comparing two different groupings produced by our model due to the difficulty of interpreting sentence embeddings produced by neural networks. Also, the Silhouette score is not an accurate estimate to calibrate the items in the identified banks of items. Thus, the quality of our banks of items needs to be validated by expert knowledge to ensure that the emerged list of items covered the construct of mobility based on the ICF categories. Regarding the items, we have not accounted for the time frame and response options while analysing the clusters, as we only accounted for the content of the item. Finally, we note that, while our procedure was retained for its overall simplicity, other alternatives exist for sentence clustering. These alternatives are however out of scope of the current paper due to the large time consumption involved in evaluating another clustering by experts.

## Conclusion

The comprehensive banks of items of mobility presented in this study has multiple uses: First, it represents a first step toward establishing a comprehensive COS and a common language of mobility among individuals with ABI to develop the ontology. Second, it enables researchers and healthcare professionals to begin exposing the content of mobility measures as a way to assess mobility comprehensively among individuals with ABI. Ultimately, using shared assessment items of mobility it may be possible to adapt these items across the continuum of care. Our banks of items of mobility will soon be used to develop the ontology, allowing the stakeholders to make decisions about tailored individualized treatment plans. Lastly, the promising results obtained in this study provide a road map for using NLP in other health outcome areas and we expect they will motivate future works in this direction to leverage alternative NLP techniques.

## Supplementary Information

**Additional file 1.**

## Data Availability

The findings of the detailed preliminary banks of items are available upon request. For further details regarding availability of data, please contact Dr. Sara Ahmed: Tel.: 514-398-4400 ext. 00531; E-mail: sara.ahmed@mcgill.ca

## Abbreviations

ABI: Acquired Brain Injury
BERT: Bidirectional Encoder Representations from Transformers
CAT: Computerised Adaptive Testing
COSMIN: Consensus-based Standards for the Selection of Health Measurement Instrument
COS: Core Outcome Set
GPS: Global Positioning System
ICF: International Classification, Functioning, Disability and Health
IRT: Item Response Theory
ML: Machine Learning
Neuro-QoL: Quality of Life in Neurologic Disorders
NLP: Natural Language Processing
PCA: Principal Component Analysis
PRISMA: Preferred Reporting Items for Systematic Reviews and Meta-Analyses
PRO: Patient-reported outcome
PROMIS: Patient-Reported Outcomes Measurement Information System
TBI: Traumatic Brain Injury
TBI-QoL: Traumatic Brain Injury Quality of Life

## References

[1] Maas AI, Stocchetti N, Bullock R (2008). Moderate and severe traumatic brain injury in adults. Lancet Neurol.

[2] Patil M, Gupta A, Khanna M, Taly AB, Soni A, Kumar JK (2017). Cognitive and functional outcomes following inpatient rehabilitation in patients with acquired brain injury: a prospective follow-up study. J Neurosci Rural Prac.

[3] Prabhakaran V. Non-communicable diseases in India: Transitions, burden of disease and risk factors-A short story. India Health Beat. 2012;6(1).

[4] Dewan MC, Rattani A, Gupta S, Baticulon RE, Hung Y-C, Punchak M (2018). Estimating the global incidence of traumatic brain injury. J Neurosurg.

[5] Chambers-Richards TL-TA (2020). Risk factors, trends, health care and general life satisfaction for select neurological conditions among an aging population in.

[6] Thrift AG, Thayabaranathan T, Howard G, Howard VJ, Rothwell PM, Feigin VL (2017). Global stroke statistics. Int J Stroke.

[7] Scholten AC, Haagsma JA, Andriessen T, Vos P, Steyerberg E, Van Beeck E (2015). Health-related quality of life after mild, moderate and severe traumatic brain injury: patterns and predictors of suboptimal functioning during the first year after injury. Injury..

[8] Yin S, Njai R, Barker L, Siegel PZ, Liao Y (2016). Summarizing health-related quality of life (HRQOL): development and testing of a one-factor model. Popul Health Metr.

[9] Zampolini M, Corea F, Avesani R, Boldrini P, De Tanti A, Di Stefano M (2013). Rehabilitation of acquired brain injuries: a multicentric prospective survey. Eur J Phys Rehabil Med.

[10] Feigin VL, Forouzanfar MH, Krishnamurthi R, Mensah GA, Connor M, Bennett DA (2014). Global and regional burden of stroke during 1990–2010: findings from the Global Burden of Disease Study 2010. Lancet.

[11] Peel C, Baker PS, Roth DL, Brown CJ, Bodner EV, Allman RM (2005). Assessing mobility in older adults: the UAB Study of Aging Life-Space Assessment. Phys Ther.

[12] Shumway-Cook A, Patla AE, Stewart A, Ferrucci L, Ciol MA, Guralnik JM (2002). Environmental demands associated with community mobility in older adults with and without mobility disabilities. Phys Ther.

[13] Tsai L-T. Walking, physical activity and life-space mobility among older people. Stud Sport Phys Educ Health. 2017;254.

[14] Webber SC, Porter MM, Menec VH (2010). Mobility in older adults: a comprehensive framework. Gerontologist.

[15] Baker PS, Bodner EV, Allman RM (2003). Measuring life-space mobility in community-dwelling older adults. J Am Geriatr Soc.

[16] May D, Nayak U, Isaacs B (1985). The life-space diary: a measure of mobility in old people at home. Int Rehabil Med.

[17] Stalvey BT, Owsley C, Sloane ME, Ball K (1999). The Life Space Questionnaire: A measure of the extent of mobility of older adults. J Appl Gerontol.

[18] Burns SP, Schwartz JK, Scott SL, Devos H, Kovic M, Hong I (2018). Interdisciplinary approaches to facilitate return to driving and return to work in mild stroke: a position paper. Arch Phys Med Rehabil.

[19] Organization WH (2001). International classification of functioning, disability and health: ICF.

[20] Clarke P, Ailshire JA, Bader M, Morenoff JD, House JS (2008). Mobility disability and the urban built environment. Am J Epidemiol.

[21] Nagel CL, Carlson NE, Bosworth M, Michael YL (2008). The relation between neighborhood built environment and walking activity among older adults. Am J Epidemiol.

[22] Murphy MA, Resteghini C, Feys P, Lamers I (2015). An overview of systematic reviews on upper extremity outcome measures after stroke. BMC Neurol.

[23] Miller EL, Murray L, Richards L, Zorowitz RD, Bakas T, Clark P (2010). Comprehensive overview of nursing and interdisciplinary rehabilitation care of the stroke patient: a scientific statement from the American Heart Association. Stroke..

[24] Party ISW (2012). National clinical guideline for stroke.

[25] Tsyben A, Guilfoyle M, Timofeev I, Anwar F, Allanson J, Outtrim J (2018). Spectrum of outcomes following traumatic brain injury—relationship between functional impairment and health-related quality of life. Acta Neurochir.

[26] Quatrano LA, Cruz TH (2011). Future of outcomes measurement: impact on research in medical rehabilitation and neurologic populations. Arch Phys Med Rehabil.

[27] McCulloch KL, De Joya AL, Hays K, Donnelly E, Johnson TK, Nirider CD (2016). Outcome measures for persons with moderate to severe traumatic brain injury: recommendations from the American Physical Therapy Association Academy of Neurologic Physical Therapy TBI EDGE Task Force. J Neurol Phys Ther.

[28] Horton L, Rhodes J, Wilson L (2018). Randomized controlled trials in adult traumatic brain injury: a systematic review on the use and reporting of clinical outcome assessments. J Neurotrauma.

[29] Rappaport M, Hall K, Hopkins K, Belleza T, Cope D (1982). Disability rating scale for severe head trauma: coma to community. Arch Phys Med Rehabil.

[30] Cella D, Riley W, Stone A, Rothrock N, Reeve B, Yount S (2010). The Patient-Reported Outcomes Measurement Information System (PROMIS) developed and tested its first wave of adult self-reported health outcome item banks: 2005–2008. J Clin Epidemiol.

[31] Hays RD, Spritzer KL, Amtmann D, Lai J-S, DeWitt EM, Rothrock N (2013). Upper-extremity and mobility subdomains from the Patient-Reported Outcomes Measurement Information System (PROMIS) adult physical functioning item bank. Arch Phys Med Rehabil.

[32] Cella D, Lai J-S, Nowinski C, Victorson D, Peterman A, Miller D (2012). Neuro-QOL: brief measures of health-related quality of life for clinical research in neurology. Neurology..

[33] Tulsky DS, Kisala PA (2019). An overview of the traumatic brain injury–quality of life (TBI-QOL) measurement system. J Head Trauma Rehabil.

[34] Ware JE, Bjorner JB, Kosinski M. Practical implications of item response theory and computerized adaptive testing: a brief summary of ongoing studies of widely used headache impact scales. Med Care. 2000;38(9):II73-II82. 10982092

[35] Cella D, Chang C-H. A discussion of item response theory and its applications in health status assessment. Med Care. 2000;38(9):II66-II72. 10.1097/00005650-200009002-00010 10982091

[36] Chang W-C, Chan C, Slaughter SE, Cartwright D (1997). Evaluating the FONE FIM: Part II. Concurrent validity & influencing factors. J Outcome Measure.

[37] Larsen KR, Michie S, Hekler EB, Gibson B, Spruijt-Metz D, Ahern D (2017). Behavior change interventions: the potential of ontologies for advancing science and practice. J Behav Med.

[38] Okhmatovskaia A, Shaban-Nejad A, Lavigne M, Buckeridge DL, editors. Addressing the challenge of encoding causal epidemiological knowledge in formal ontologies: a practical perspective. MIE; 2014. 25160364

[39] Andrich D (1988). Rasch models for measurement.

[40] Locoro A, Mascardi V, Scapolla AM, editors. NLP and Ontology Matching-A Successful Combination for Trialogical Learning. ICAART (1); 2010.

[41] Velupillai S, Suominen H, Liakata M, Roberts A, Shah AD, Morley K (2018). Using clinical Natural Language Processing for health outcomes research: Overview and actionable suggestions for future advances. J Biomed Inform.

[42] Le Q, Mikolov T, editors. Distributed representations of sentences and documents. International conference on machine learning; 2014: PMLR.

[43] Alhasani R, Auger C, Paiva Azevedo M, Ahmed S. Quality of mobility measures among individuals with acquired brain injury: an umbrella review. Qual Life Res. 2022. 10.1007/s11136-022-03103-4 PMC9356944 35275377

[44] Mokkink LB, Prinsen C, Patrick DL, Alonso J, Bouter LM, de Vet H (2018). COSMIN methodology for systematic reviews of patient-reported outcome measures (PROMs). User Manual.

[45] Alhasani R, Radman D, Auger C, Lamontagne A, Ahmed S (2021). Clinicians and individuals with acquired brain injury perspectives about factors that influence mobility: creating a core set of mobility domains among individuals with acquired brain injury. Ann Med.

[46] Mark Vrabel MLS. Preferred reporting items for systematic reviews and meta-analyses. In: Oncology nursing forum. Oncology Nursing Society. 2015. p. 552.‏ 10.1188/15.ONF.552-554 26302284

[47] Reimers N, Gurevych I. Sentence-bert: Sentence embeddings using siamese bert-networks. arXiv preprint arXiv:190810084. 2019.

[48] Har-Peled S, Indyk P, Motwani R (2012). Approximate nearest neighbor: Towards removing the curse of dimensionality. Theory Computing.

[49] Syms C (2019). Principal Components Analysis.

[50] McInnes L, Healy J, Melville J. Umap: Uniform manifold approximation and projection for dimension reduction. arXiv preprint arXiv:180203426. 2018.

[51] Pedregosa F, Varoquaux G, Gramfort A, Michel V, Thirion B, Grisel O (2011). Scikit-learn: Machine learning in Python. J Machine Learn Res.

[52] Lloyd S (1982). Least squares quantization in PCM. IEEE Trans Inform Theory.

[53] Lee JA, Verleysen M (2007). Nonlinear dimensionality reduction.

[54] Rousseeuw PJ (1987). Silhouettes: a graphical aid to the interpretation and validation of cluster analysis. J Computation Appl Math.

[55] Goutte C, Gaussier E, editors. A probabilistic interpretation of precision, recall and F-score, with implication for evaluation. In: European conference on information retrieval. Berlin, Heidelberg: Springer; 2005. p. 345-359.‏

[56] Uzuner Ö, South BR, Shen S, DuVall SL (2011). 2010 i2b2/VA challenge on concepts, assertions, and relations in clinical text. J Am Med Inform Assoc.

[57] Ashford S, Brown S, Turner-Stokes L (2015). Systematic review of patient-reported outcome measures for functional performance in the lower limb. J Rehabil Med.

[58] Ashford S, Slade M, Malaprade F, Turner-Stokes L (2008). Evaluation of functional outcome measures for the hemiparetic upper limb: a systematic review. J Rehabil Med.

[59] Baker K, Cano SJ, Playford ED (2011). Outcome measurement in stroke: a scale selection strategy. Stroke..

[60] Barak S, Duncan PW (2006). Issues in selecting outcome measures to assess functional recovery after stroke. NeuroRx..

[61] Connell LA, Tyson SF (2012). Clinical reality of measuring upper-limb ability in neurologic conditions: a systematic review. Arch Phys Med Rehabil.

[62] Croarkin E, Danoff J, Barnes C (2004). Evidence-based rating of upper-extremity motor function tests used for people following a stroke. Phys Ther.

[63] Fini NA, Holland AE, Keating J, Simek J, Bernhardt J (2015). How is physical activity monitored in people following stroke?. Disabil Rehabil.

[64] Gebruers N, Vanroy C, Truijen S, Engelborghs S, Deyn D (2010). Monitoring of physical activity after stroke: a systematic review of accelerometry-based measures. Arch Phys Med Rehabil.

[65] Geroin C, Mazzoleni S, Smania N, Gandolfi M, Bonaiuti D, Gasperini G (2013). Systematic review of outcome measures of walking training using electromechanical and robotic devices in patients with stroke. J Rehabil Med.

[66] Geyh S, Kurt T, Brockow T, Cieza A, Ewert T, Omar Z, et al. Identifying the concepts contained in outcome measures of clinical trials on stroke using the International Classification of Functioning, Disability and Health as a reference. J Rehabil Med 2004;36(0):56-62. 10.1080/16501960410015399 15370749

[67] Gor-García-Fogeda MD, Molina-Rueda F, Cuesta-Gómez A, Carratalá-Tejada M, Alguacil-Diego IM, Miangolarra-Page JC (2014). Scales to assess gross motor function in stroke patients: a systematic review. Arch Phys Med Rehabil.

[68] Hong I, Bonilha HS (2017). Psychometric properties of upper extremity outcome measures validated by Rasch analysis: a systematic review. Int J Rehabil Res.

[69] Lemmens RJ, Timmermans AA, Janssen-Potten YJ, Smeets RJ, Seelen HA (2012). Valid and reliable instruments for arm-hand assessment at ICF activity level in persons with hemiplegia: a systematic review. BMC Neurol.

[70] Lord SE, Rochester L (2005). Measurement of community ambulation after stroke: current status and future developments. Stroke..

[71] Martins JC, Aguiar LT, Nadeau S, Scianni AA, Teixeira-Salmela LF, Faria CDCDM (2019). Measurement properties of self-report physical activity assessment tools for patients with stroke: a systematic review. Braz J Phys Ther.

[72] McCabe P, Lippert C, Weiser M, Hilditch M, Hartridge C, Villamere J (2007). Community reintegration following acquired brain injury. Brain Inj.

[73] Mudge S, Stott NS (2007). Outcome measures to assess walking ability following stroke: a systematic review of the literature. Physiotherapy..

[74] Nichol AD, Higgins A, Gabbe B, Murray L, Cooper D, Cameron P (2011). Measuring functional and quality of life outcomes following major head injury: common scales and checklists. Injury..

[75] Oczkowski C, O'Donnell M (2010). Reliability of proxy respondents for patients with stroke: a systematic review. J Stroke Cerebrovasc Dis.

[76] Pearson OR, Busse M, Van Deursen RWM, Wiles CM (2004). Quantification of walking mobility in neurological disorders. Qjm..

[77] Pollock C, Eng J, Garland S (2011). Clinical measurement of walking balance in people post stroke: a systematic review. Clin Rehabil.

[78] Rowland TJ, Gustafsson L (2008). Assessments of upper limb ability following stroke: a review. Br J Occupation Therapy.

[79] Salbach NM, O’brien KK, Brooks D, Irvin E, Martino R, Takhar P (2017). Considerations for the selection of time-limited walk tests poststroke: a systematic review of test protocols and measurement properties. J Neurol Phys Ther.

[80] Salter K, Jutai J, Teasell R, Foley N, Bitensky J (2005). Issues for selection of outcome measures in stroke rehabilitation: ICF Body Functions. Disabil Rehabil.

[81] Salter K, Jutai J, Teasell R, Foley N, Bitensky J, Bayley M (2005). Issues for selection of outcome measures in stroke rehabilitation: ICF Participation. Disabil Rehabil.

[82] Salter K, Jutai J, Teasell R, Foley N, Bitensky J, Bayley M (2005). Issues for selection of outcome measures in stroke rehabilitation: ICF activity. Disabil Rehabil.

[83] Scrivener K, Sherrington C, Schurr K (2013). A systematic review of the responsiveness of lower limb physical performance measures in inpatient care after stroke. BMC Neurol.

[84] Silva PF, Quintino LF, Franco J, Faria CD (2014). Measurement properties and feasibility of clinical tests to assess sit-to-stand/stand-to-sit tasks in subjects with neurological disease: a systematic review. Braz J Phys Ther.

[85] Simpson LA, Eng JJ (2013). Functional recovery following stroke: capturing changes in upper-extremity function. Neurorehabil Neural Repair.

[86] Sivan M, O'Connor RJ, Makower S, Levesley M, Bhakta B (2011). Systematic review of outcome measures used in the evaluation of robot-assisted upper limb exercise in stroke. J Rehabil Med.

[87] Sorrentino GSP, Solaro C, Rabini A, Cerri C, Ferriero G. Clinical measurement tools to assess trunk performance after stroke: a systematic review. Eur J Phys Rehabil Med. 2018. 10.23736/S1973-9087.18.05178-X 29684980

[88] Steins D, Dawes H, Esser P, Collett J. Wearable accelerometry-based technology capable of assessing functional activities in neurological populations in community settings: a systematic review. J Neuroeng Rehabil 2014;11(1):1-13. 10.1186/1743-0003-11-36 PMC4007563 24625308

[89] Stevens PM (2010). Clinimetric properties of timed walking events among patient populations commonly encountered in orthotic and prosthetic rehabilitation. J Prosthetics Orthotics.

[90] Teale EA, Young JB (2010). A review of stroke outcome measures valid and reliable for administration by postal survey. Rev Clin Gerontol.

[91] Tse T, Douglas J, Lentin P, Carey L (2013). Measuring participation after stroke: a review of frequently used tools. Arch Phys Med Rehabil.

[92] Tyson S, Connell L (2009). The psychometric properties and clinical utility of measures of walking and mobility in neurological conditions: a systematic review. Clin Rehabil.

[93] van Bloemendaal M, van de Water AT, van de Port IG (2012). Walking tests for stroke survivors: a systematic review of their measurement properties. Disabil Rehabil.

[94] Van Peppen RP, Hendriks H, Van Meeteren NL, Helders PJ, Kwakkel G (2007). The development of a clinical practice stroke guideline for physiotherapists in The Netherlands: a systematic review of available evidence. Disabil Rehabil.

[95] Velstra I-M, Ballert CS, Cieza A (2011). A systematic literature review of outcome measures for upper extremity function using the international classification of functioning, disability, and health as reference. PM&R..

[96] Verceles AC, Hager ER (2015). Use of accelerometry to monitor physical activity in critically ill subjects: a systematic review. Respir Care.

[97] Verheyden G, Nieuwboer A, Van de Winckel A, De Weerdt W (2007). Clinical tools to measure trunk performance after stroke: a systematic review of the literature. Clin Rehabil.

[98] Wang Q, Markopoulos P, Yu B, Chen W, Timmermans A (2017). Interactive wearable systems for upper body rehabilitation: a systematic review. J Neuroeng Rehabil.

[99] Wang S, Hsu CJ, Trent L, Ryan T, Kearns NT, Civillico EF (2018). Evaluation of performance-based outcome measures for the upper limb: a comprehensive narrative review. PM&R..

[100] Wilde EA, Whiteneck GG, Bogner J, Bushnik T, Cifu DX, Dikmen S (2010). Recommendations for the use of common outcome measures in traumatic brain injury research. Arch Phys Med Rehabil.

[101] Williams G, Robertson V, Greenwood K (2004). Measuring high-level mobility after traumatic brain injury. Am J Phys Med Rehabil.

[102] Zheng H, Black ND, Harris ND (2005). Position-sensing technologies for movement analysis in stroke rehabilitation. Med Biol Eng Comput.

[103] Rose M, Bjorner JB, Becker J, Fries J, Ware J (2008). Evaluation of a preliminary physical function item bank supported the expected advantages of the Patient-Reported Outcomes Measurement Information System (PROMIS). J Clin Epidemiol.

[104] Al Zoubi F, Mayo N, Rochette A, Thomas A (2018). Applying modern measurement approaches to constructs relevant to evidence-based practice among Canadian physical and occupational therapists. Implement Sci.

[105] Tsekleves E, Skordoulis D, Paraskevopoulos I, Kilbride C, Warland A, editors. Personalised stroke rehabilitation intervention using open source 3D software and the Wii Remote Plus. Proc 9th Intl Conf Disabil Virtual Real Assoc Technol, Laval, France; 2012.

[106] Boulkedid R, Abdoul H, Loustau M, Sibony O, Alberti C (2011). Using and reporting the Delphi method for selecting healthcare quality indicators: a systematic review. PLoS One.

[107] Hasson F, Keeney S, McKenna H (2000). Research guidelines for the Delphi survey technique. J Adv Nurs.

[108] Murry JW, Hammons JO (1995). Delphi: A versatile methodology for conducting qualitative research. Rev Higher Educ.

[109] Liang W, Zou J, Yu Z. Alice: Active learning with contrastive natural language explanations. arXiv preprint arXiv:200910259. 2020.

[110] Fries J, Ramey D (1993). Platonic outcomes. J Rheumatol.

[111] Ware J, Kosinski M, Bjorner J (2004). Item banking and the improvement of health status measures. Qual Life.

[112] Ware JE, Kosinski M, Bjorner JB, Bayliss MS, Batenhorst A, Dahlöf CG (2003). Applications of computerized adaptive testing (CAT) to the assessment of headache impact. Qual Life Res.

[113] Cella D, Lai J (2004). Core item banking program: Past, present and future. Qual Life Res.

[114] Heifetz RA, Heifetz R, Grashow A, Linsky M (2009). The practice of adaptive leadership: Tools and tactics for changing your organization and the world.

