# Supplementary material for: Informing the development of an outcome set and banks of items to measure mobility among individuals with acquired brain injury using natural language processing
Source: BMC Neurol. 2022 Dec 9;22:464. doi: 10.1186/s12883-022-02938-1 (PMC9733317; doi:10.1186/s12883-022-02938-1)
Supplement: Supplementary file 1 — Additional file 1. [file 12883_2022_2938_MOESM1_ESM.docx]

**Appendix 1: The Microsoft office Excel 2010 functionalities**

| **Task** | **Functionalities** |
| --- | --- |
| 2.1. Export to .CSV file | All columns and rows were visible and no blank rows appeared within the range. |
| 2.2. Remove duplicate rows | Removing duplicate rows function. |
| 2.3. Correct spelling mistakes | Spell-check and grammar functions. |
| 2.4. Changing the case | The “LOWER” functions in Excel that converts all uppercase letters in a text string to lowercase letters. |
| 2.5. Extend acronyms and abbreviations to their full form | Excel functions “FIND, SEARCH, REPLACE, SUBSTITUTE, LEFT, RIGHT” that can perform various string manipulation tasks, such as finding and replacing a sub-string within a string, extracting portions of a string, or determining the length of a string. |
| 2.6. Fixing numbers and number signs | The “VALUE” functions in Excel to convert a text string that representing a number to a number format. |
| 2.7. Remove white spaces, non-printing characters, typos, punctuations from the sentence | the “SUBSTITUTE” function to replace one character in a text string with a different character. |
